# Beyond the Mandibular Canal: A Narrative Review of Inferior Alveolar Nerve Variations and Their Clinical Significance

**DOI:** 10.7759/cureus.116431

**Published:** 2026-09-17

**Authors:** Evgenia Moschogianni, George Triantafyllou, Alexandros Samolis, Dimosthenis Chrysikos, Nikolaos Kolomvos, Nikolaos Lazaridis, Maria Piagkou

**Affiliations:** 1 Department of Anatomy, School of Medicine, Faculty of Health Sciences, National and Kapodistrian University of Athens, Athens, GRC; 2 Department of Oral and Maxillofacial Surgery, School of Dentistry, Faculty of Health Sciences, National and Kapodistrian University of Athens, Athens, GRC; 3 Department of Anatomy and Surgical Anatomy, School of Medicine, Faculty of Health Sciences, Aristotle University of Thessaloniki, Thessaloniki, GRC

**Keywords:** anatomical variation, auriculotemporal nerve, craniofacial surgery, inferior alveolar nerve, lingual nerve, local anesthesia, mandibular canal, mandibular nerve, mylohyoid nerve, oral surgery

## Abstract

The inferior alveolar nerve (IAN) is commonly presented as a relatively constant branch of the mandibular nerve that gives rise to the mylohyoid nerve (MHN), enters the mandibular foramen, follows a single mandibular canal, and terminates as mental and incisive branches. However, direct human anatomical evidence shows substantially greater complexity. This critical narrative review synthesizes cadaveric, microdissection, histological, imaging, and osteological evidence on proximal IAN roots, relationships with the maxillary artery, communications with neighboring mandibular nerve branches, MHN-derived accessory pathways, intramandibular fascicular organization, and neural structures associated with accessory canals or foramina. PubMed/MEDLINE was searched through August 20, 2026, with focused supplementary searches, Google Scholar citation searching, and reference-list screening; direct human neural evidence was prioritized over osseous inference. Histological studies show that a grossly single IAN can contain separately organized neural components, whereas proximal dissections demonstrate double roots, artery-through-nerve configurations, recurrent branches, and communications with the lingual and auriculotemporal nerves. The MHN may extend beyond its conventional motor territory, communicate with the lingual nerve, and use accessory mandibular pathways. Conversely, an accessory foramen or canal does not by itself establish the presence of an accessory IAN branch because some channels are predominantly vascular. The evidence is therefore best interpreted as a branching neuro-osseous continuum rather than a canal-only model. Clinically, these variants may help explain selected patterns of incomplete anesthesia or unexpected sensory and surgical anatomy, but bony imaging alone should not be used to infer causation.

## Introduction and background

The inferior alveolar nerve (IAN) is classically described as a major sensory branch of the posterior division of the mandibular nerve (V3). It descends through the infratemporal fossa (ITF), usually gives rise to the mylohyoid nerve (MHN) before the mandibular foramen (MBF), and then accompanies the inferior alveolar vessels within the mandible (Figure 1).

**Figure 1:**
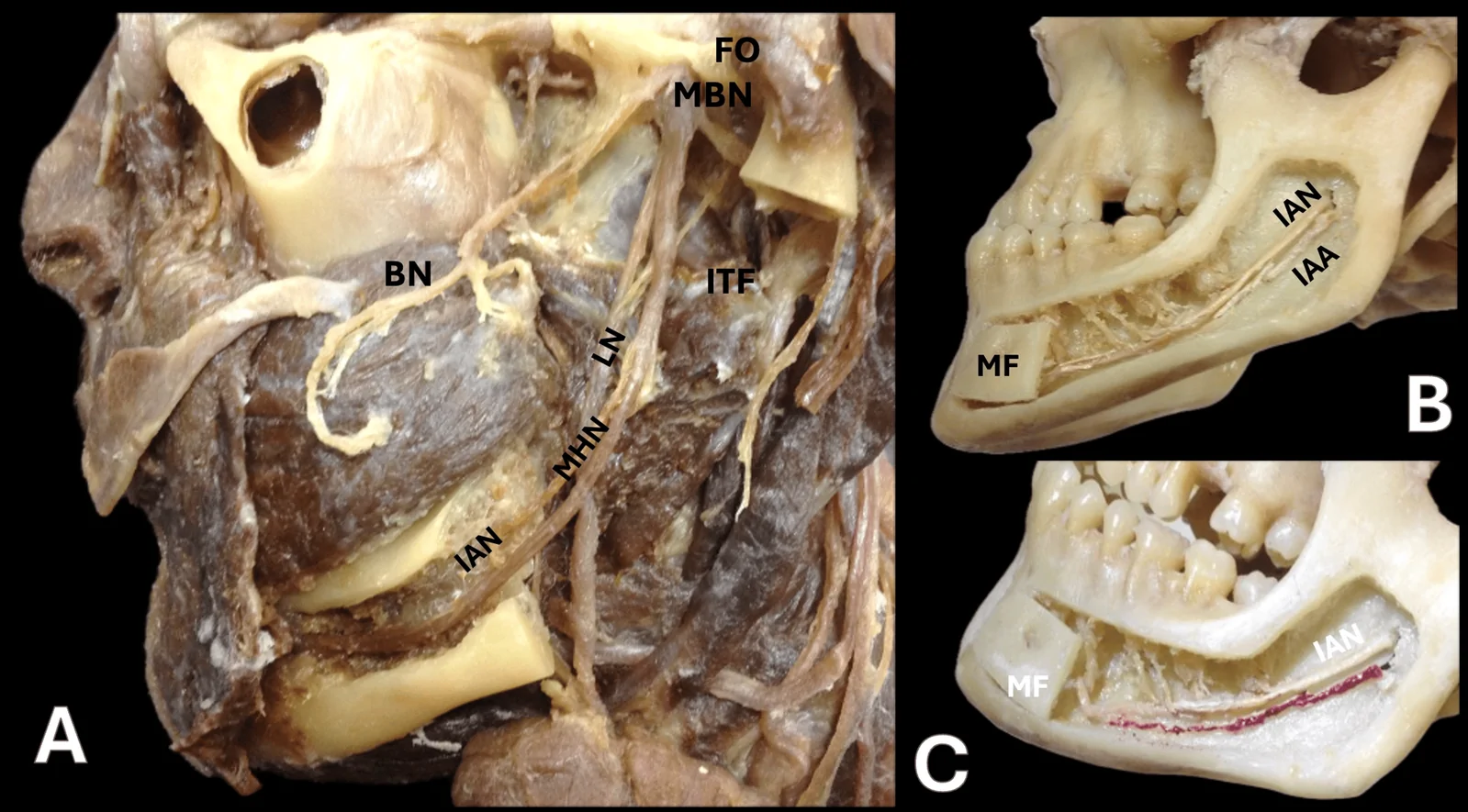
Cadaveric demonstration of the extra- and intramandibular course of the inferior alveolar neurovascular bundle. (A) Dissection of the infratemporal fossa (ITF) demonstrating the mandibular nerve (MBN) emerging from the foramen ovale (FO) and its relationship with the buccal nerve (BN), lingual nerve (LN), inferior alveolar nerve (IAN), and mylohyoid nerve (MHN). (B) Lateral view of a dissected mandible after removal of the lateral cortical plate, exposing the intramandibular course of the inferior alveolar nerve (IAN) and inferior alveolar artery (IAA) within the mandibular canal toward the mental foramen (MF). (C) A further dissected specimen demonstrating the close parallel relationship between the IAN and IAA within the mandibular canal and their course toward the MF. Abbreviations: BN, buccal nerve; FO, foramen ovale; IAA, inferior alveolar artery; IAN, inferior alveolar nerve; ITF, infratemporal fossa; LN, lingual nerve; MBN, mandibular nerve; MF, mental foramen; MHN, mylohyoid nerve. Image Credit: Prof. Dr. Maria Piagkou. Original unpublished cadaveric dissections and photographs prepared by the authors.

Textbook descriptions commonly reduce its intramandibular course to a single trunk that gives dental branches and divides near the mental foramen (MF) into mental and incisive components. Human dissection and histology show that this model is useful for orientation but incomplete for describing neural architecture [1-4].

Wadu et al. showed that the radiographic mandibular canal does not necessarily represent a continuous cortical tube and that the main nerve may separate into mental and incisive components in the molar region, well before the MF [1]. Kqiku et al. demonstrated histologically that a grossly single IAN contains two larger neural components with separate perineurial sheaths and a changing relationship along the mandibular body [2]. Lee et al. independently confirmed a major mental-dental organization and showed that anterior dental fascicles may continue through intertrabecular spaces where a discrete corticated incisive canal is not evident [3]. In a larger microdissection series of 107 edentulous mandibles, Kieser et al. documented multiple intrabony distribution patterns, including a single trunk with simple superior branches and a molar plexus [4]. These studies show that canal appearance does not fully represent the underlying neural organization.

Variation also occurs before mandibular entry, beyond the field represented by routine canal imaging. Direct human studies describe double or multiple IAN roots, relationships in which the maxillary artery (MA) passes between or through neural components, recurrent accessory branches, plexiform arrangements, and communications with the lingual nerve (LN), auriculotemporal nerve (ATN), MHN, and other V3 branches [5-20]. Distally, the MHN can continue beyond its conventional muscular territory and participate in sensory or neurovascular pathways associated with lingual, retromental, or midline foramina [21-29].

Most contemporary radiological studies are canal-centered because cone-beam computed tomography (CBCT) depicts bone well and permits reproducible classification of bifid or trifid canals and accessory mandibular or mental foramina [30-34]. They define an osseous phenotype, not neural architecture itself. An accessory channel may contain a nerve, artery, vein, or mixed neurovascular bundle, while a soft-tissue neural communication may leave no distinct bony signature.

This review therefore focuses on the relationship between direct neural anatomy and its osseous correlates rather than on another prevalence summary of mandibular canal morphology. Variation is considered by anatomical level: proximal root formation, neurovascular relationships, communications among posterior V3 branches, MHN accessory pathways, intramandibular fascicular organization, and terminal or accessory foraminal routes. Detailed morphometry of the anterior loop of the mental nerve and conventional distal mental nerve branching patterns was outside the primary scope unless directly relevant to an accessory MF or another noncanonical IAN pathway. In this context, the IAN is more accurately viewed as a variable branching neuro-osseous system than as a single nerve confined to one canal.

## Review

Literature search approach

We conducted a targeted PubMed/MEDLINE search to identify human anatomical evidence relevant to IAN variation. The original search was completed on August 20, 2026, and was updated on September 14, 2026. The updated strategy comprised one principal search and three focused supplementary searches.

Principal Search

("inferior alveolar nerve"[Title/Abstract] OR "inferior dental nerve"[Title/Abstract]) AND (anatom*[Title/Abstract] OR morpholog*[Title/Abstract] OR variat*[Title/Abstract] OR branch*[Title/Abstract] OR bifurcat*[Title/Abstract] OR root*[Title/Abstract] OR fascicul*[Title/Abstract] OR communication*[Title/Abstract] OR "maxillary artery"[Title/Abstract] OR "lingual nerve"[Title/Abstract] OR "auriculotemporal nerve"[Title/Abstract] OR "mylohyoid nerve"[Title/Abstract] OR "mandibular canal"[Title/Abstract] OR "accessory mandibular foramen"[Title/Abstract] OR "accessory mental foramen"[Title/Abstract] OR "retromolar canal"[Title/Abstract] OR "incisive canal"[Title/Abstract] OR "lingual canal"[Title/Abstract]).

Supplementary Search 1: IAN Communications/Root Patterns

("inferior alveolar nerve"[Title/Abstract] OR "inferior dental nerve"[Title/Abstract]) AND ("lingual nerve"[Title/Abstract] OR "auriculotemporal nerve"[Title/Abstract]) AND (communication*[Title/Abstract] OR root*[Title/Abstract] OR branch*[Title/Abstract] OR anastomos*[Title/Abstract]).

Supplementary Search 2: Mylohyoid Accessory Pathways

"mylohyoid nerve"[Title/Abstract] AND (accessory[Title/Abstract] OR sensory [Title/Abstract] OR dental[Title/Abstract] OR innervation[Title/Abstract] OR communication*[Title/Abstract] OR foramen[Title/Abstract] OR foramina[Title/Abstract] OR canal[Title/Abstract]).

Supplementary Search 3: Osseous Correlates

("mandibular canal"[Title/Abstract] OR "bifid mandibular canal"[Title/Abstract] OR "trifid mandibular canal"[Title/Abstract] OR "accessory mandibular foramen"[Title/Abstract] OR "accessory mandibular foramina"[Title/Abstract] OR "accessory mental foramen"[Title/Abstract] OR "accessory mental foramina"[Title/Abstract] OR "retromolar canal"[Title/Abstract] OR "incisive canal"[Title/Abstract] OR "lingual canal"[Title/Abstract] OR "lingual canals"[Title/Abstract]) AND ("inferior alveolar"[Title/Abstract] OR mylohyoid[Title/Abstract] OR nerve[Title/Abstract] OR neural[Title/Abstract] OR neurovascular[Title/Abstract]).

No language filter was applied to the updated PubMed search. Non-English studies were eligible when their anatomical findings could be adequately evaluated from the abstract, full text, or an available translation. Google Scholar citation searching and reference-list screening were also used, particularly for older anatomical reports that may be incompletely indexed in PubMed. Records retrieved by more than one search were consolidated manually by title, authorship, and publication details and were considered once in the synthesis; formal dual-reviewer deduplication was not performed.

Human cadaveric dissection, microdissection, histology, immunohistochemistry, and studies directly tracing canal or foraminal contents were given the greatest interpretive weight because they can demonstrate neural continuity or tissue identity. Imaging and dry-bone studies, systematic reviews, and meta-analyses were used mainly to characterize osseous correlates and clinical context. Case reports were retained when they provided direct, anatomically informative observations of uncommon neural configurations or canal/foraminal contents that were not adequately represented in larger series; they were used for anatomical characterization and hypothesis generation, not for prevalence estimation. Animal studies and studies focused only on nerve-injury treatment, routine third-molar outcomes, automated canal segmentation, or conventional mandibular morphometry without information on neural variation were not used as primary anatomical evidence.

This was a narrative anatomical review rather than a systematic prevalence review. We did not perform PRISMA (Preferred Reporting Items for Systematic Reviews and Meta-Analyses) screening, duplicate independent study selection, formal risk-of-bias scoring, or quantitative pooling. Study inclusion was based on anatomical relevance and evidentiary value, and numerical frequencies are reported only for individual studies. Clinical claims were interpreted according to what the underlying method could establish, rather than formally graded: direct anatomy establishes continuity and topography, histology confirms tissue identity, functional studies support physiological relevance, and clinical causation requires patient-level evidence.

Why a canal-centered model is incomplete

The MBC is the bony environment of a neurovascular bundle, not a direct image of IAN’s internal organization. Wadu et al. examined 29 human mandibles radiographically and then decalcified and dissected nine. Canal borders ranged from apparently continuous radiopaque lines to poorly defined or interrupted outlines. Dissection showed that surrounding trabeculae accounted for much of this radiographic appearance; the bundle was surrounded by bone but was not consistently enclosed within a uniform cortical tube [1].

In the dissected specimens, the main nerve divided into mental and incisive components in the molar region. A molar branch arose soon after canal entry; the mental and incisive components then ran in close parallel, with small communications more evident anteriorly. Premolar innervation was plexiform in several specimens, and a recurrent contribution from the mental nerve re-entered the mandible in the canine-first premolar region [1]. Thus, division of the IAN may occur well before the MF.

Kqiku et al. examined 20 partially dentulous hemimandibles, using 10 for gross dissection and 10 for histological analysis. In the histological specimens, the IAN contained two larger neural components, each enclosed by a separate perineurial sheath and both surrounded by a common epineurium. The dental component consisted of several smaller bundles and continued anteriorly as the incisive nerve, whereas the mental component formed a larger bundle and changed position relative to the dental component along the canal, producing a spiraling relationship rather than simple parallelism [2].

Lee et al. examined 20 dentulous hemimandibles histologically. The neurovascular bundle contained a large nerve trunk composed mainly of mental and dental components. The mental component lay inferolingually in the third-molar region, turned buccally near the first molar, and separated from the dental component before the MF. In the lateral-incisor region, small dental fascicles and arteries occupied intertrabecular spaces even when a distinct corticated incisive canal was not present [3]. Absence of a discrete anterior canal on imaging therefore does not exclude anterior neural tissue.

Variability is also evident at the macroscopic level. Kieser et al. microdissected 107 edentulous mandibles and found that the most frequent pattern was a single intrabony trunk with a series of simple branches directed toward the alveolar crest; the next most common was a small nerve plexus in the molar region [4]. Although edentulous anatomy is influenced by alveolar resorption, the study demonstrates that intrabony distribution is not reducible to a single branching pattern.

Terms such as "single," "bifid," or "trifid IAN" require anatomical qualification. A grossly single trunk may contain separately organized fascicular components, while distinct neural branches may share one osseous corridor or occupy different canals. The radiographic canal maps the bony neurovascular pathway but does not define fascicular organization, fiber modality, or inter-nerve connectivity.

Proximal root variations and the MA

Proximal variation occurs between V3 and the MBF, outside the segment represented by routine MBC imaging. Wolf et al. illustrated bilateral splitting of the IAN around the MA in a cadaver, with an additional IAN-LN communication on one side, emphasizing that neural and vascular variants can coexist [5]. Larger primary anatomical series, however, show that the artery-nerve relationship is not limited to isolated cases [6-9].

Pretterklieber et al. reinvestigated the MA in 102 individuals. Among several infratemporal patterns, the MA passed medial to the lateral pterygoid muscle and pierced the IAN in 4.9% of specimens [6]. The observation in a series of this size shows that this artery-nerve relationship is not confined to isolated case reports.

Roy et al. examined 40 cadaveric heads (80 sides) and described an IAN arising by two long roots from the posterior division of V3. The roots reunited to form a single trunk, while the second part of the MA passed through the neural loop. The MHN arose from the deep root before reunion [7]. Khan et al. documented a related but morphologically distinct configuration in 50 ITF: in three fossae (6%), the MA passed through the IAN, separating it into superficial and deep portions that reunited below the vessel [8]. Thus, an artery lying between primary IAN roots should be distinguished from an artery traversing a secondary divided IAN trunk.

Buch and Agnihotri identified another proximal pattern in 35 dissected sides. A recurrent variant branch arose from the IAN just below the MHN origin in 12/35 sides (34.3%). In some specimens it terminated in the lateral pterygoid, while in others it passed through the muscle to join the anterior or posterior division of V3 or one of their branches, leading the authors to suggest that some configurations could be interpreted as an additional IAN root [9]. The finding further blurs the boundary between “root,” “branch,” and “communication.”

Developmental explanations for these variants commonly invoke persistence or remodeling of alternative relationships among growing mandibular nerve fibers, masticatory muscle primordia, and the first-arch vascular plexus [7-9]. Such mechanisms are anatomically plausible, but they should remain hypotheses: serial human embryological evidence directly tracing these specific configurations into adult anatomy is limited.

Clinically, proximal variants are relevant to high mandibular blocks, ITF procedures, nerve repair, skull-base surgery, and operations in which the MA and posterior V3 branches are exposed. Vascular pulsation or tethering within a neural loop has been proposed as a possible contributor to selected pain syndromes [7,8], but these anatomical observations do not establish a causal relationship with neuralgia.

The IAN as part of a communicating V3 network

IAN-LN Communications

Communications between the IAN and LN are among the most repeatedly described posterior V3 variations. Rácz and Maros reported one or several connecting bridges between the LN and IAN in 25% of 48 head halves, while a related study by Rácz, Maros, and Seres-Sturm identified IAN-LN bridges in 16.6% of 18 human heads [10,11]. These historical studies used heterogeneous terminology and should not be pooled, but they establish that IAN-LN communication is not restricted to isolated case reports.

Sakamoto and Akita examined 24 head halves from 12 cadavers and described frequent direct IAN-LN communications proximal to the MHN origin. They also observed branches from the LN entering the mylohyoid muscle and communicating with the proper MHN [12]. Kim et al. performed macroscopic and microscopic dissection in 32 Korean hemi-heads and identified several communication patterns: four specimens had MHN-LN communications, four had IAN-ATN communications, and additional specimens showed IAN communication with the nerve to the lateral pterygoid or related motor pathways. Two specimens displayed a plexiform V3 branching arrangement [13].

These studies describe the posterior division of V3 as a variably communicating system: the IAN, LN, ATN, MHN, and selected motor branches may be linked by neural twigs rather than remain completely separate. The presence of a communication, however, does not establish the direction of conduction or the sensory, motor, or autonomic composition of its fibers.

IAN-ATN Communications and Root Contributions

IAN-ATN relationships form a continuum from discrete communicating twigs to roots that participate directly in ATN formation. Anıl et al. found variant anatomy in two of 20 infratemporal dissections. In one cadaver, a branch from the ATN joined the IAN bilaterally; the resulting neural loop was closely related to the MA, and histological sections confirmed true neural tissue with myelinated fibers [14].

Thotakura et al. examined 36 infratemporal regions and described several posterior-division variants, including an ATN-IAN communication that formed a buttonhole configuration for the MHN and another arrangement with two communicating branches from the lower ATN root to the IAN [15]. Bhardwaj et al. described a bilateral IAN-ATN communication in a cadaveric series and performed histology demonstrating epineurial, perineurial, and fascicular organization within the communicating branch [16].

Komarnitki et al. classified the ATN according to its root system in 80 specimens, including 40 adult and 40 fetal sides. ATN roots arose either from the MBN alone or with contributions from the IAN, including configurations associated with a bifurcated IAN [17]. Similar patterns in fetal material are compatible with a developmental origin and also explain why comparable structures have been termed an ATN root, connecting branch, or IAN-ATN communication.

Tritsch et al. reported a histologically examined infratemporal communication interpreted as passing from the IAN toward the ATN [18]. Tsakiris et al. later described a cadaver with a branch between one of three ATN roots and the IAN on one side and three separate IAN-LN communications on the other [19]. Different communication patterns may therefore coexist in the same individual.

Moodley et al. examined 32 cadaveric specimens. ATN root number ranged from one to four; 18/32 ATNs originated from the mandibular nerve without an IAN contribution, whereas 14/32 had at least one communication with the IAN [20]. With the adult and fetal observations of Komarnitki et al. [17], this indicates that IAN participation in ATN root architecture is a recurring morphological pattern.

Morphological Communication Does Not Automatically Demonstrate Functional Collateral Innervation

Direct visualization of a communicating neural branch establishes anatomical continuity but does not automatically establish functional collateral innervation. Gross anatomy cannot determine whether a connection is predominantly sensory, motor, autonomic, or mixed, and routine histology is more reliable for confirming neural tissue than for defining fiber modality or direction. Histochemical, immunohistochemical, electrophysiological, or tracing approaches provide stronger functional evidence, but such methods remain uncommon in human IAN literature [14,16,18,24].

This limitation matters when anatomical variants are used to explain anesthetic failure or neuropathic symptoms. Collateral pathways could contribute to residual sensation, but block success is also affected by injection level, technical accuracy, inflammation, anesthetic concentration and volume, fascial barriers, and individual tissue characteristics. Neural communications should therefore be described primarily as anatomical findings unless functional or patient-level evidence supports a clinical mechanism [5,24,29].

The MHN as an Accessory Sensory and Neuro-Osseous Pathway

The MHN is usually described as a predominant motor branch that leaves the IAN before the MBF and supplies the mylohyoid muscle and anterior belly of the digastric. Human evidence shows variability in its level and site of origin, communications, terminal sensory distribution, and relationship with accessory foramina [21-29].

Madeira et al. dissected 26 adult cadavers and found a supplementary MHN-derived branch entering the mandible through accessory foramina on the lingual side of the symphysis in 13 cases (50%). The branch most often entered the inferior retromental foramen and, when traced intraosseously, could terminate in incisor teeth or gingiva or join the ipsilateral or contralateral incisive nerve [21]. This directly links an MHN branch with an accessory osseous route and the anterior dental neural system.

Wilson et al. examined 37 adult cadavers and found that the MHN branched 5-23 mm before the MBF, with a mean distance of 14.7 mm. In 16/37 cases (43%), the nerve extended beyond the MHM toward foramina on the lingual aspect of the mandible [22]. Bennett and Townsend traced the MHN from the ITF to its terminal branches in six cadavers and demonstrated variability in branch level, passage through a mylohyoid groove or canal, muscular branches, and terminal submental branching; fine branches entered foramina in the canine or premolar region in some specimens [23].

Zhan et al. examined MHN-LN communications in 62 embalmed and 16 fresh hemifaces. Nineteen of the 62 embalmed specimens had a communication that pierced the MHM, and histochemical staining supported a sensory component [24]. Unlike purely descriptive dissections, this study provided evidence about fiber phenotype in at least some MHN-LN communications.

Koga et al. examined 440 sides from 303 elderly Japanese cadavers using microscopic and immunohistochemical methods. Communication between the MHN and LN was identified in 62.5% (275/440) of sides, and calcitonin gene-related peptide-positive structures were present within the mylohyoid fascia [25]. The findings document frequent communication in this sample but do not by themselves establish a specific pain mechanism.

Nayak and Soumya described an MHN arising from the IAN within the mandible and exiting through a separate mylohyoid foramen before reaching the mylohyoid and anterior digastric [26]. In 40 infratemporal sides, Chandrasekaran et al. found three variant MHN patterns (7.5%): two arose directly from the mandibular nerve and one from the medial aspect of the IAN [27]. The conventional sequence of a precanalicular MHN arising from the IAN is therefore not universal.

Liang et al. combined morphometric examination of 50 dry mandibles with macro- and microanatomical dissection of 12 intact cadaveric mandibles. The superior and inferior midline lingual canals contained different neurovascular structures, and a branch of the MHN entered the inferior genial spinal canal [28]. This provides direct correlation between an osseous canal and MHN-derived neural tissue.

Sillanpää et al. directed local anesthetic toward the MHN in volunteers and reported partial anesthesia in 21% of subjects, which they interpreted as evidence of sensory input to the first mandibular molar in some individuals [29]. The observation is limited and should not be generalized, but it is compatible with the anatomical evidence for sensory fibers in selected MHN pathways (Table 1).

**Table 1:** Representative direct human anatomical and histological evidence on inferior alveolar nerve variation. Frequencies are shown only to characterize individual studies and are not pooled prevalence estimates. Abbreviations: ATN, auriculotemporal nerve; IAN, inferior alveolar nerve; LN, lingual nerve; MHN, mylohyoid nerve; V3, mandibular division of the trigeminal nerve.

Study	Material/evidence modality	Principal observation	Interpretive value	What the evidence cannot establish
Wadu et al. [1]	Radiography followed by decalcification and dissection	Mental-incisive separation occurred in the molar region; recurrent mental branch and anterior plexiform patterns were observed.	Directly separates neural branching from the apparent radiographic canal.	Fiber modality, physiological conduction, or clinical causation.
Kqiku et al. [2]	Gross dissection + histology	Two major neural components had separate perineurial sheaths within a common epineurium and changed relative position along the canal.	Histological evidence that a single IAN is internally compartmentalized.	Functional direction of fibers or clinical consequences of the compartments.
Lee et al. [3]	Histology	Mental and dental components separated before the MF; anterior dental fascicles could occupy intertrabecular spaces without a discrete corticated incisive canal.	Demonstrates that osseous canal appearance does not fully map neural extent.	Functional conduction, symptoms, or anesthetic relevance in individual patients.
Kieser et al. [4]	Microdissection	Single-trunk and molar-plexus intrabony patterns were common.	Large direct series showing variable intramandibular distribution.	Fiber modality or clinical causation; edentulous anatomy may not represent dentulous adults.
Pretterklieber et al. [6]	Infratemporal dissection	Maxillary artery pierced IAN in 4.9%.	Series-level documentation of nerve-artery interdigitation.	Whether the relationship produces symptoms or alters nerve function.
Roy et al. [7]	Cadaveric dissection	Double-rooted IAN with maxillary artery between roots; MHN arose from the deep root.	Direct proximal root and neurovascular anatomy.	Functional significance or population-wide prevalence beyond the sampled material.
Khan et al. [8]	Infratemporal dissection	The maxillary artery passed through the IAN in 3/50 fossae, and the neural portions reunited below it.	Series-level confirmation of an artery-through-nerve configuration.	Neural fiber modality or causal association with pain/neuropathy.
Buch & Agnihotri [9]	Cadaveric dissection	Recurrent IAN branch in 12/35 sides; some branches rejoined V3 divisions and could be interpreted as accessory roots.	Bridges the descriptive concepts of root, branch, and communication.	Direction of signaling, fiber type, or clinical effect.
Kim et al. [13]	Macroscopic and microscopic dissection	Multiple communication patterns involving MHN-LN, IAN-ATN, and IAN-motor branches; plexiform V3 in two specimens.	Documents multiple communication patterns within posterior V3.	Fiber modality, direction of transmission, or functional collateral innervation.
Anıl et al. [14]	Dissection + histology	Bilateral ATN-IAN communication in one cadaver; neural tissue confirmed histologically.	Confirm that the communicating structure was neural.	Sensory/motor/autonomic composition, conduction direction, or clinical causation.
Komarnitki et al. [17]	Adult and fetal cadaveric dissection	ATN roots could include contributions from the IAN; comparable configurations occurred in fetal material.	Developmental and terminological framework for ATN-IAN continuity.	Direct embryological lineage, fiber modality, or functional significance.
Moodley et al. [20]	Cadaveric dissection	ATN had 1–4 roots; 14/32 had at least one IAN communication.	Contemporary series supporting recurrent IAN contribution to ATN root architecture.	Functional transmission or symptom causation.
Madeira et al. [21]	Cadaveric dissection with intraosseous tracing	The MHN-derived branch entered the retromental/lingual foramina in 13/26 and could join incisive nerves or reach teeth/gingiva.	Direct nerve-to-bone-to-dental pathway.	Fiber modality and the magnitude of its functional contribution in living patients.
Wilson et al. [22]	Cadaveric dissection	MHN branched 5–23 mm before the MBF; 43% extended toward lingual foramina.	Demonstrates that the MHN may arise substantially proximally to the MBF and continue toward accessory lingual routes.	Whether these branches provide effective dental sensation or cause anesthetic failure.
Zhan et al. [24]	Microdissection + histochemical analysis	19/62 embalmed specimens had MHN-LN communication through the mylohyoid; staining supported a sensory character.	Stronger human evidence for sensory fibers in some MHN-LN communications.	Specific target territory, conduction direction, or clinical block failure.
Liang et al. [28]	Dry-bone morphometry + macro/microdissection	The inferior genial spinal canal contained a branch derived from the MHN.	Direct osseous-neural correlation.	Functional importance or prevalence of the same content in all similar canals.
Kodera & Hashimoto [33]	Anatomical case study/direct canal-content tracing	The retromolar canal contained an IAN-derived retromolar nerve and an arterial branch.	Direct evidence that some accessory canals contain true IAN branches.	Population prevalence or generalizability from a single case.
Iwanaga et al. [34]	Fresh-frozen dissection with vascular tracing	Contents traced through accessory mandibular foramina were predominantly vascular in this series.	Shows that an accessory foramen does not necessarily represent an accessory IAN branch.	Whether foramina at other locations contain nerves or mixed bundles.
Przystańska & Bruska [35]	Cadaveric dissection + histology + immunohistochemistry	Accessory foramina on the medial mandibular body contained neurovascular bundles; histology and neural markers confirmed an accompanying nerve.	Location-specific confirmation that some accessory mandibular foramina contain both arterial and neural components.	Precise neural fiber modality, functional conduction, or clinical causation.
Toh et al. [36]	Cadaveric anatomical study (three cases)	Accessory mental nerves emerged through accessory mental foramina and showed variable peripheral distributions and communications.	Direct anatomical evidence that some accessory mental foramina transmit neural branches.	Prevalence, population generalizability, fiber modality, or clinical causation.

Osseous correlates: when bone reflects and does not reflect the nerve

The inferior alveolar neurovascular structures develop in close spatial relation to the mandible so that some neural branches may have an osseous correlate. The most informative studies are those that directly traced or histologically examined canal or foraminal contents rather than inferring them from imaging alone.

Kodera and Hashimoto described a large retromolar canal arising from the MBC that contained an arterial branch from the inferior alveolar artery and a retromolar nerve from the IAN trunk. The nerve supplied the mandibular third molar, retromolar mucosa, buccal mucosa, and buccal gingiva of the premolar-molar region [33]. This is a directly traced IAN branch within an accessory osseous canal.

Liang et al. demonstrated neurovascular content in midline lingual canals and an MHN branch within the inferior genial spinal canal [28]. Madeira et al. likewise traced MHN-derived branches through retromental foramina toward anterior dental pathways [21]. These observations confirm that some accessory canals correspond to neural branches.

The relationship is not one-to-one. Iwanaga et al. dissected 22 fresh-frozen sides to investigate accessory mandibular foramina around the MBF. The traced contents in their series were vascular, consisting of branches of the MA or tributaries of the inferior alveolar or maxillary venous systems [34]. An accessory MBF therefore cannot be assumed to transmit an accessory to the IAN branch.

Przystańska and Bruska examined accessory foramina on the medial mandibular body by dissection, histology, and immunohistochemistry. They identified neurovascular bundles containing an artery accompanied by a nerve, with positive neural markers [35]. Their findings complement the predominantly vascular contents reported by Iwanaga et al. and show that foraminal contents vary with location.

Toh et al. examined accessory mental foramina and their nerves in three cadavers. Accessory mental nerves emerged through the foramina and showed variable peripheral distributions, including communications with facial and buccal nerve branches [36]. The series is too small for prevalence estimates but directly demonstrates neural contents in accessory mental foramina.

Population imaging and osteological studies describe the bony phenotype. Asghar et al. summarized the frequency and morphology of bifid and trifid mandibular canals [30]. Thomaidi and Papadopoulos combined dry-mandible anatomy with meta-analysis of accessory mandibular foramina [31], and Thomaidi et al. reviewed accessory mental foramina as terminal osseous pathways [32]. Przystańska and Bruska and Toh et al. add direct tissue evidence for neural contents in selected accessory mandibular and mental foramina [35,36]. Imaging and dry-bone studies, however, report skeletal routes and should not be read as prevalence estimates of independently verified neural branches.

The methods are not interchangeable. Dissection defines topography; histology or immunohistochemistry can confirm tissue identity and may sometimes indicate fiber phenotype; imaging identifies an osseous route but usually not the contents of a small channel or a soft-tissue communication; and dry-bone anatomy demonstrates only a potential neurovascular corridor. Clinical interpretation should remain within those limits.

An Extreme Example of Nerve-Bone Developmental Coupling

Manikandhan et al. described a young woman with hemifacial microsomia in whom the IAN entered the mandible at an unusually high level, traversed only the width of the ramus, and exited laterally rather than continuing through the mandibular body. The ipsilateral mandibular canal and MF were absent [37]. The variant was confirmed surgically and therefore provides an unusual direct example of an altered neural course accompanied by altered osseous development.

This congenital case cannot be generalized to normal adult variation, but it shows that a major change in IAN trajectory may coexist with altered mandibular canal and foraminal development. It illustrates developmental coupling between nerve and bone without implying that every additional canal contains an additional nerve.

Clinical implications

IAN Block

Anatomical variation may contribute to incomplete IAN block in selected patients, but it is only one of several possible explanations. Failure may also reflect injection technique, landmark localization, inflammation, anesthetic concentration or volume, diffusion barriers, fascial anatomy, and individual physiological response. Anatomical variants are therefore most useful as an explanation for persistent or anatomically patterned failure after common technical causes have been considered, rather than as a default explanation for every unsuccessful block.

Several mechanisms are anatomically credible. A high or multiple IAN root configuration may lie proximal to the conventional deposit; an MHN may leave the IAN 5-23 mm before the MBF [22]; MHN-derived fibers may enter through independent lingual or retromental foramina [21,28]; and communications among IAN, LN, and ATN may provide collateral routes [10-20]. Selective MHN anesthesia producing partial molar anesthesia in some volunteers provides limited functional support for an accessory dental contribution [29].

When technically adequate conventional blocks repeatedly remain incomplete, these anatomical variants may justify consideration of a more proximal mandibular block or another appropriate supplemental technique. They do not support routine additional injections, and a bifid canal or accessory foramen on CBCT should not be assigned causal significance without concordant clinical findings.

Third-Molar, Retromolar, and Mandibular Ramus Surgery

Posterior mandibular procedures occur near the IAN, LN, retromolar neural twigs, the MHN branching zone, and the MA. Kim et al. documented frequent collateral LN twigs to the retromolar region together with several V3 communication patterns [13], while Kodera and Hashimoto directly demonstrated an IAN-derived retromolar nerve within an accessory canal [33]. These pathways may be encountered even when the dominant mandibular canal appears conventionally.

During third-molar surgery, sagittal split osteotomy, ramus osteotomy, nerve repair, or infratemporal dissection, risk is therefore not confined to the largest radiographically visible canal. Accessory branches, unusual proximal roots, or altered artery-nerve relationships may occupy adjacent tissue planes. Conversely, accessory foramina around the ramus may be vascular rather than neural [34], so their operative significance may include hemorrhagic as well as sensory risk.

Implant Placement and Anterior Mandibular Surgery

Early separation of the mental and incisive components has practical implications for implant placement, periapical surgery, genioplasty, chin bone harvesting, nerve lateralization, and repair. Wadu et al., Kqiku et al., and Lee et al. demonstrate that neural separation may occur posterior to the MF and that anterior dental fascicles can persist even when a discrete bony incisive canal is not apparent [1-3]. Surgical planning should therefore respect the expected neural territory rather than rely exclusively on the visible canal lumen.

Anterior accessory pathways are also relevant. MHN-derived branches may enter through retromental or midline lingual foramina and join incisive pathways [21,28], while accessory mental foramina may transmit terminal neurovascular structures. Toh et al. directly traced accessory mental nerves through such foramina, whereas imaging and osteological studies mainly define the bony phenotype [32,36]. Cross-sectional imaging should be interpreted in that context and should not be obtained solely to search for theoretical neural variants.

Craniofacial Pain and Neuropathy

Abnormal IAN-MA relationships and IAN-ATN communications have been proposed as potential contributors to referred or neuropathic symptoms in selected individuals [6-9,14-20]. Such mechanisms are anatomically plausible when a nerve is traversed, tethered, or incorporated into a vascular or neural loop, but most supporting studies are anatomical and were not designed to establish symptom causality.

Unusual anatomy is therefore a differential consideration, not a primary explanation for nonspecific facial pain. Clinical attribution requires concordance with the symptom distribution, exclusion of common dental and temporomandibular causes, and, when appropriate, imaging or operative evidence.

Imaging Interpretation: Limitations and Capabilities of CBCT

CBCT depicts cortical and cancellous bone well and is useful when clinically indicated for implant planning, impacted teeth, mandibular surgery, or characterization of accessory canals. It does not, however, directly resolve IAN fascicular architecture or most soft-tissue communications among V3 branches.

Wadu et al. showed that an apparently continuous canal border may reflect trabecular architecture rather than a true cortical sleeve [1]. Lee et al. also demonstrated anterior neural fascicles within intertrabecular spaces when a discrete corticated incisive canal was absent [3]. The radiographic canal is therefore a proxy for the neurovascular pathway, not a complete map of IAN architecture.

A bifid or trifid canal indicates a branched osseous neurovascular route, not a specific accessory sensory nerve. An accessory channel may contain a nerve, artery, vein, or combination, while neural communications may occur without a separate canal. Imaging should remain indication-driven and should not be used as a stand-alone map of neural connectivity.

A Hierarchical Framework for IAN Variation

Table 2 organizes the evidence by anatomical level so that the site of a variant is separated from the strength and type of evidence supporting its clinical interpretation. It is a descriptive framework, not a validated classification.

**Table 2:** Anatomical level-based descriptive framework for inferior alveolar nerve variations and their clinical relevance. This framework is a clinically oriented descriptive synthesis and is not a validated formal classification. Abbreviations: ATN, auriculotemporal nerve; IAN, inferior alveolar nerve; LN, lingual nerve; MHN, mylohyoid nerve; V3, mandibular division of the trigeminal nerve.

Anatomical level	Representative variation	Strongest supporting evidence	Potential clinical relevance
I. Proximal origin	Double/multiple roots; recurrent accessory root-like branch; plexiform V3	Wolf [5]; Roy [7]; Buch [9]; Kim [13]	High-level anesthesia; infratemporal and skull-base surgical anatomy
II. Neurovascular relationship	Maxillary artery between roots or piercing IAN	Pretterklieber [6]; Roy [7]; Khan [8]	Unexpected artery-nerve proximity; bleeding risk; possible tethering
III. IAN-LN network	Single or multiple communications	Rácz [10,11]; Sakamoto [12]; Kim [13]; Tsakiris [19]	Potential collateral pathways; variable operative anatomy
IV. IAN-ATN network	Communicating twig or ATN root contribution from IAN	Anıl [14]; Thotakura [15]; Bhardwaj [16]; Komarnitki [17]; Moodley [20]	Posterior V3 surgical anatomy; pain attribution remains uncertain
V. Mylohyoid pathway	High origin, direct V3 origin, LN communication, sensory terminal branch, intraosseous origin	Madeira [21]; Wilson [22]; Zhan [24]; Nayak [26]; Chandrasekaran [27]	Potential accessory dental or gingival sensation; possible incomplete block in selected cases
VI. Mandibular entry/accessory foramina	Separate entry or accessory osseous pathway; neural or vascular content	Madeira [21]; Liang [28]; Iwanaga [34]; Przystańska & Bruska [35]; Thomaidi [31]	Additional operative corridor; neural versus vascular content must not be assumed
VII. Intramandibular organization	Early mental-incisive separation; plexiform dental branches; separate fascicular compartments	Wadu [1]; Kqiku [2]; Lee [3]; Kieser [4]	Implant, osteotomy, periapical, and nerve-repair planning
VIII. Terminal/retromolar pathways	Retromolar nerve; accessory mental exit; incisive cross-connections	Kodera [33]; Wadu [1]; Thomaidi et al. [32]; Toh et al. [36]	Third-molar, retromolar, and anterior mandibular procedures

A Branching Neuro-Osseous Continuum Rather Than Isolated Anomalies

The reported variants extend from the ITF to terminal mental, incisive, retromolar, and mylohyoid-related pathways. Proximal variation involves root number, recurrent branches, vascular interdigitation, and communications with neighboring V3 branches. The MHN may carry sensory fibers, communicate with the LN, or follow an accessory osseous route. Within the mandible, a grossly single IAN may contain separately organized components and may divide earlier than textbook descriptions suggest. Distally, accessory mental, incisive, retromolar, and lingual pathways provide additional exits or cross-connections.

Some variants are entirely soft-tissue and may have no CBCT correlate; others are associated with a canal or foramen. Neural branching, vascular branching, and mandibular ossification are related, but the relationship is not one-to-one. Imaging therefore depicts only part of the anatomy.

For clinical use, anatomical level and tissue content may matter more than prevalence alone. A rare proximal root configuration can be relevant during an infratemporal procedure, while a small MHN-derived pathway may matter in a patient with repeated anesthetic failure.

What this review adds

The review brings together evidence that is often discussed separately: proximal IAN root variation, artery-nerve relationships, communications among posterior V3 branches, MHN-derived pathways, intramandibular fascicular organization, and the actual contents of accessory osseous routes. A canal-centered classification captures only part of this anatomy.

Three practical points follow. Neural and osseous variation should be correlated but not equated. Roots, recurrent branches, communicating twigs, accessory branches, and terminal plexuses should be described by anatomical level before a clinical mechanism is proposed. Finally, the strength of a clinical claim should match the evidence: dissection establishes anatomical possibility, histology confirms tissue identity, functional studies address physiological relevance, and causation requires patient-level concordance.

This approach allows classic cadaveric observations and modern imaging findings to be considered together without treating them as methodologically equivalent.

Limitations of the evidence and future directions

Several limitations constrain the evidence base. Many direct anatomical observations come from small cadaveric series or single cases, and specimen age, dentition, fixation, side, ancestry, and disease status are inconsistently reported. Older studies also use heterogeneous denominators, including heads, sides, nerves, hemimandibles, or whole mandibles, which limits direct comparison of reported frequencies.

Terminology is inconsistent. The same structure may be labeled a root, branch, communicating twig, accessory nerve, or recurrent branch depending on the direction of dissection and the author's interpretation. Komarnitki et al. illustrated this problem for the ATN root system [17]. A clearer descriptive vocabulary should distinguish proximal root formation, inter-nerve communication, accessory terminal branch, and osseous canal content.

Functional inference remains another limitation. Gross anatomy and routine histology can confirm that a structure is neural but usually cannot establish direction of transmission or fiber modality. Histochemical work such as that of Zhan et al. provides stronger evidence for sensory fibers [24], but such methods are uncommon. A bony canal cannot establish neural content without direct anatomical or high-resolution soft-tissue correlation.

The search was narrative rather than systematic. PubMed/MEDLINE was supplemented by three focused searches, Google Scholar citation searching, and reference-list screening, but records were consolidated manually, and we did not use protocol registration, independent duplicate screening, a validated risk-of-bias tool, or quantitative synthesis. The review is therefore an anatomical synthesis of heterogeneous evidence rather than an exhaustive prevalence estimate, and poorly indexed reports may have been missed.

Future work should combine pre-dissection CBCT or high-resolution magnetic resonance neurography with microdissection, histology or immunohistochemistry, and three-dimensional reconstruction in the same specimens. Accessory canals and foramina should be correlated with their neural and vascular contents, parent branch, direction, caliber, and target territory. Prospective clinical-anatomical studies could then test whether specific variants predict anesthetic failure, sensory disturbance, or operative risk.

## Conclusions

The IAN is more variable than the conventional model of a single nerve within a single mandibular canal suggests. Human anatomical studies document variation in proximal roots, relationships with the MA, recurrent branches, communications with the lingual and ATNs, mylohyoid pathways, intramandibular fascicular organization, and terminal distribution. Some of these neural variants have an osseous correlate; other accessory canals or foramina are vascular or mixed. A branching neuro-osseous model therefore fits the available evidence better than a canal-only model. Clinical significance should be assigned according to the anatomical level, the method used to demonstrate the variant, and the procedure involved, rather than inferred from a bony finding alone.
